# Disseminated Lodderomyces elongisporus and Pantoea dispersa: A Rare Dual Infection in an Immunocompromised Patient

**DOI:** 10.7759/cureus.58985

**Published:** 2024-04-25

**Authors:** Ee-Ming Vania San, Salina Mohamed Sukur, Ahneez Abdul Hameed, Anuradha P. Radhakrishnan

**Affiliations:** 1 Department of Internal Medicine, Hospital Selayang, Selayang, MYS; 2 Bacteriology Unit, Infectious Disease Research Centre, Institute for Medical Research, National Institutes of Health, Shah Alam, MYS; 3 Department of Pathology, Hospital Selayang, Selayang, MYS; 4 Department of Infectious Diseases, Hospital Selayang, Selayang, MYS

**Keywords:** pantoea dispersa, parapneumonic effusion, liver abscess, bacteremia, fungemia, lodderomyces elongisporus

## Abstract

With the advancement of modern medicine and the prolonged survival of critically ill patients, unusual organisms are increasingly emerging. Initially found in the environment, these rare organisms started presenting as human pathogens, causing significant morbidity and mortality. Here, we present a rare case of disseminated *Lodderomyces elongisporus* fungemia and *Pantoea dispersa* bacteremia in a patient with parapneumonic effusion and ruptured liver abscess. This yeast was identified using matrix-assisted laser desorption/ionization time-of-flight (MALDI-TOF). Although this organism has no antifungal breakpoint, the isolate shows low minimum inhibitory concentration (MIC) to a wide range of antifungals. The importance of effective communication between microbiologists and clinicians and early referral to the infectious disease team was also highlighted in this case for prompt treatment.

## Introduction

*Lodderomyces elongisporus*, formerly known as *Saccharomyces elongisporus*, was originally discovered in California citrus concentrate and South African soil in 1952 by Recca and Mrak [1]. It was later proposed in 1966 by van der Walt [1] that this yeast be classified under a new genus, as it differed significantly in morphology and physiology from the *Saccharomyces* group. Later on, researchers found similarities between *Lodderomyces elongisporus* and *Candida parapsilosis*, leading to the suggestion that it is a teleomorph of *C. parapsilosis* [2]. Older yeast identification systems such as VITEK had many times misidentified it as *C. parapsilosis*, resulting in the underreporting of cases [3,4]. In recent years, we have observed a rising number of *Lodderomyces elongisporus* cases ranging from catheter-related bloodstream infection to infective endocarditis, among others, due to the improvement of yeast identification systems [5]. On the other hand, *Pantoea dispersa* is a gram-negative bacteria found in the natural environment with agricultural and industrial benefits [6]. Although rare, there have been reports of *Pantoea dispersa* being harmful to humans and plants [6]. Here, we present a case of disseminated *Lodderomyces elongisporus* fungemia and *Pantoea dispersa* bacteremia in a patient with parapneumonic effusion and a ruptured liver abscess.

## Case presentation

A 74-year-old male presented to the emergency department with fever and epigastric pain for one week. He was a chronic smoker of 40 pack-years and was diagnosed with a pancreatic tumor five months before this presentation. It was radiologically suspicious of pancreatic carcinoma; however, biopsy samples were unsatisfactory to confirm the suspicion. An elective date for a distal pancreatectomy and splenectomy was arranged, but unfortunately, he became ill before the planned date. He also had a history of category 2 COVID-19 infection six months ago and was on home quarantine. He was not on any steroids or immunomodulators at that time. Since then, he developed a long-standing cough but did not seek medical attention for it.

**Figure 1:**
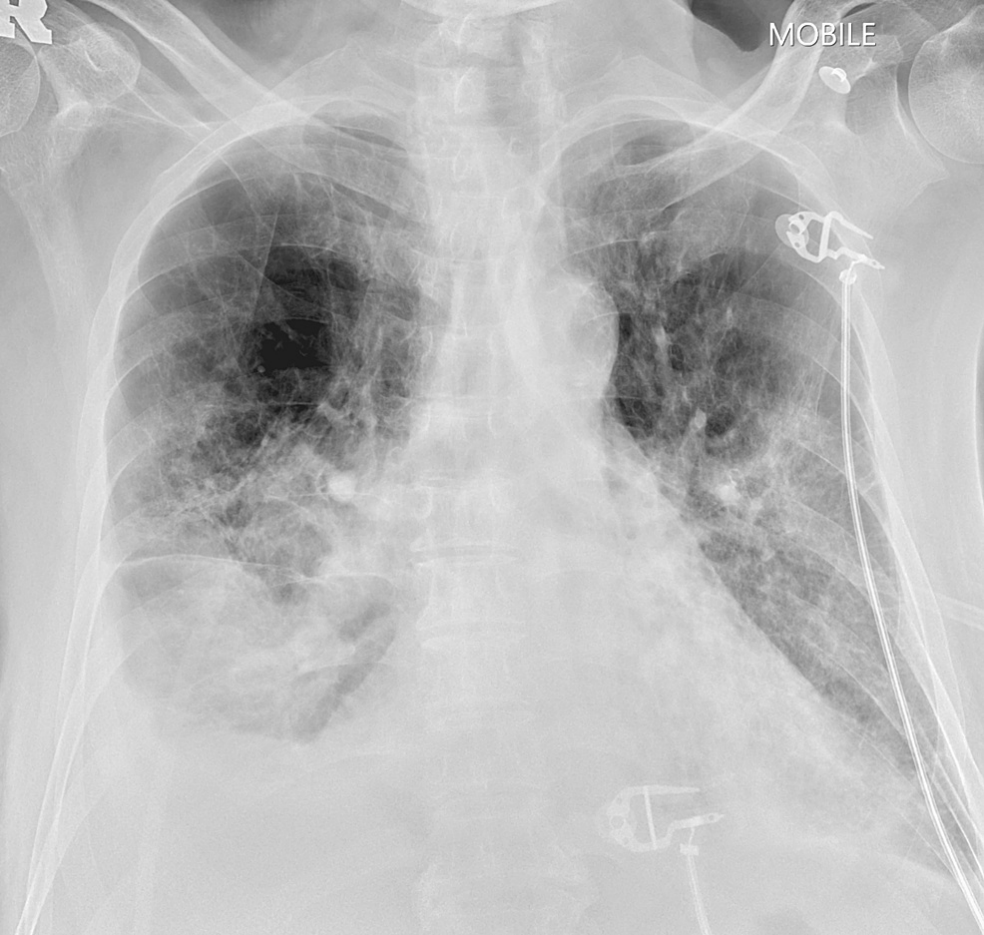
Chest X-ray showing bilateral lower zone consolidations with right pleural effusion

On arrival at the emergency department, he was in decompensated shock with a blood pressure of 89/57 mmHg, a pulse rate of 140 bpm, and a low-grade fever of 37.8°C. He was also tachypneic with a respiratory rate of 26 breaths per minute, and his oxygen saturation was 90% under room air. The cardiac monitor showed atrial fibrillation with rapid ventricular response, but otherwise, there were no murmurs heard on cardiovascular examination. He had reduced air entry over bilateral lower zones on lung auscultation, and there was tenderness of the epigastric region on palpation. His laboratory results showed elevated inflammatory markers (white blood cells, C-reactive protein, and lactate dehydrogenase level) and liver enzymes, while his arterial blood gas revealed type 1 respiratory failure with compensated metabolic acidosis (Table 1). Screening for human immunodeficiency virus, hepatitis B, hepatitis C, and rapid plasma reagin were all negative. The patient was also checked for atypical pneumonia, but serum mycoplasma and legionella serology were negative. A chest radiograph was performed and showed bilateral lower zone consolidations (Figure 1). A blood culture was obtained, and he was empirically started on intravenous ceftriaxone and azithromycin. Eventually, he was intubated in the emergency department for respiratory distress and started on inotropic support. He was then transferred to the intensive care unit for further management.

**Table 1:** Patient's initial laboratory results paO2: partial pressure of oxygen, pCO2: partial pressure of carbon dioxide

Laboratory parameters	Value	Reference range
Hemoglobin	13.2 g/dL	13-17 g/dL
White blood cell	28.7 × 10³/µL	4-10 × 10³/µL
Platelet count	296 × 10³/µL	150-410 × 10³/µL
Sodium	128 mmol/L	136-146 mmol/L
Potassium	3.1 mmol/L	3.4-4.5 mmol/L
Urea	6.3 mmol/L	2.8-7.2 mmol/L
Creatinine	95 µmol/L	64-104 µmol/L
Albumin	34 g/L	35-52 g/L
Aspartate transaminase	176 U/L	0-50 U/L
Alanine transaminase	190 U/L	0-50 U/L
Bilirubin	20 µmol/L	5-21 µmol/L
C-reactive protein	36 mg/dL	0-0.5 mg/dL
Lactate dehydrogenase	1,048 U/L	0-247 U/L
pH	7.59	7.35-7.45
paO2	53 mmHg	83-108 mmHg
pCO2	20 mmHg	35-48 mmHg
Oxygen saturation	90%	94%-98%
Bicarbonate	19.2 mmol/L	21- 28 mmol/L
Base excess	-2.5 mmol/L	-2-3 mmol/L

**Figure 2:**
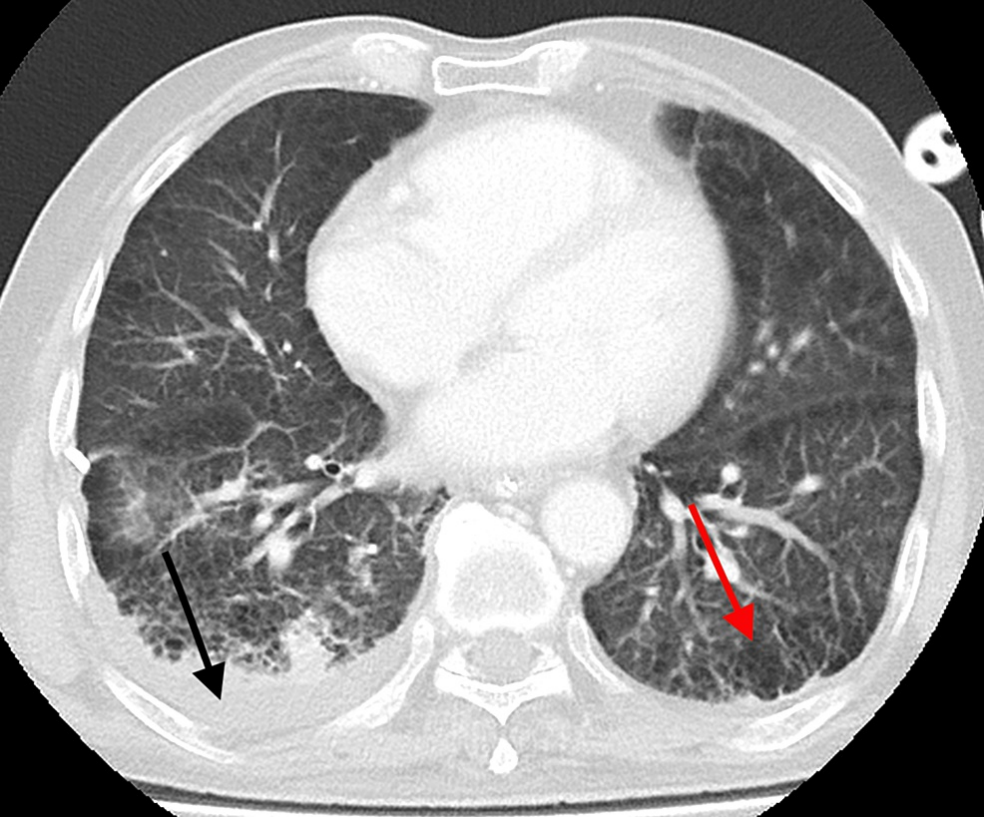
Axial view of the computed tomography of the thorax showing loculated right pleural effusion (black arrow) and emphysematous changes (red arrow)

On day 2 of admission, his blood culture grew *Pantoea dispersa*, and hence, the antibiotic was immediately upgraded to piperacillin-tazobactam as per culture and sensitivity results. Other culture results showed a mixed growth in his trachea aspirate and *Candida* species in his urine culture. As he was persistently tachycardic, a computed tomography pulmonary angiogram was arranged on day 3 of admission to look for pulmonary embolism. The scan showed no pulmonary embolism, but there was extensive bilateral pulmonary emphysema with bilateral pleural effusion, loculated on the right side (Figure 2). An ultrasound-guided right pleural drainage was done the next day and confirmed an exudative pleural effusion, but further analysis was negative for infective cultures and malignancy. On day 8 of admission, a repeated blood culture was done. It was flagged as yeast the following day, and he was started on intravenous fluconazole. The infectious disease team was consulted on day 12 of admission, and his antifungal was upgraded to anidulafungin while awaiting the identification of the yeast and its sensitivity testing. An ultrasound of the abdomen and echocardiogram were arranged on day 13 of admission to look for abscesses and vegetation. The ultrasound showed a large focal liver mass with complex perihepatic free fluid suspicious of a ruptured mass. His echocardiogram showed no vegetation, but his ejection fraction was only 30%. There was no evidence of endophthalmitis on ophthalmology assessment. A surveillance blood culture was also obtained on the same day.

**Figure 3:**
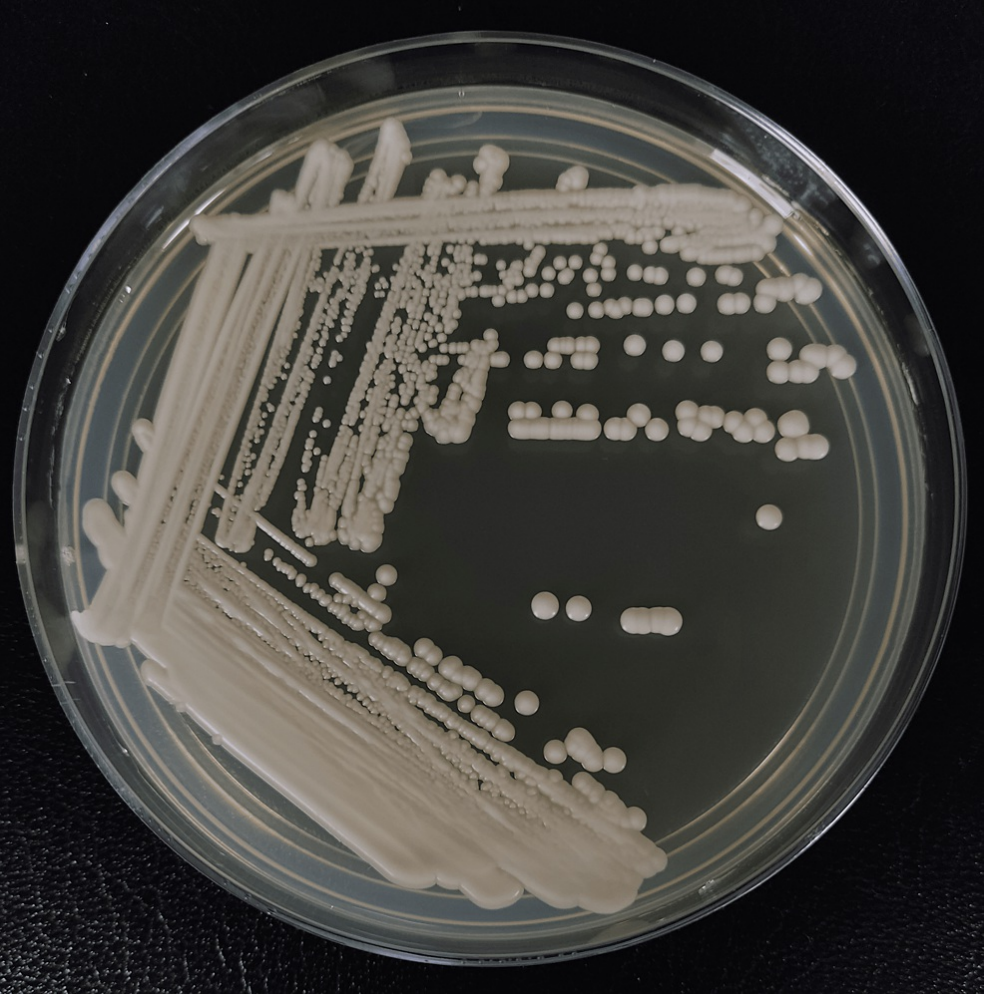
Sabouraud dextrose agar with cream-colored colonies

**Figure 4:**
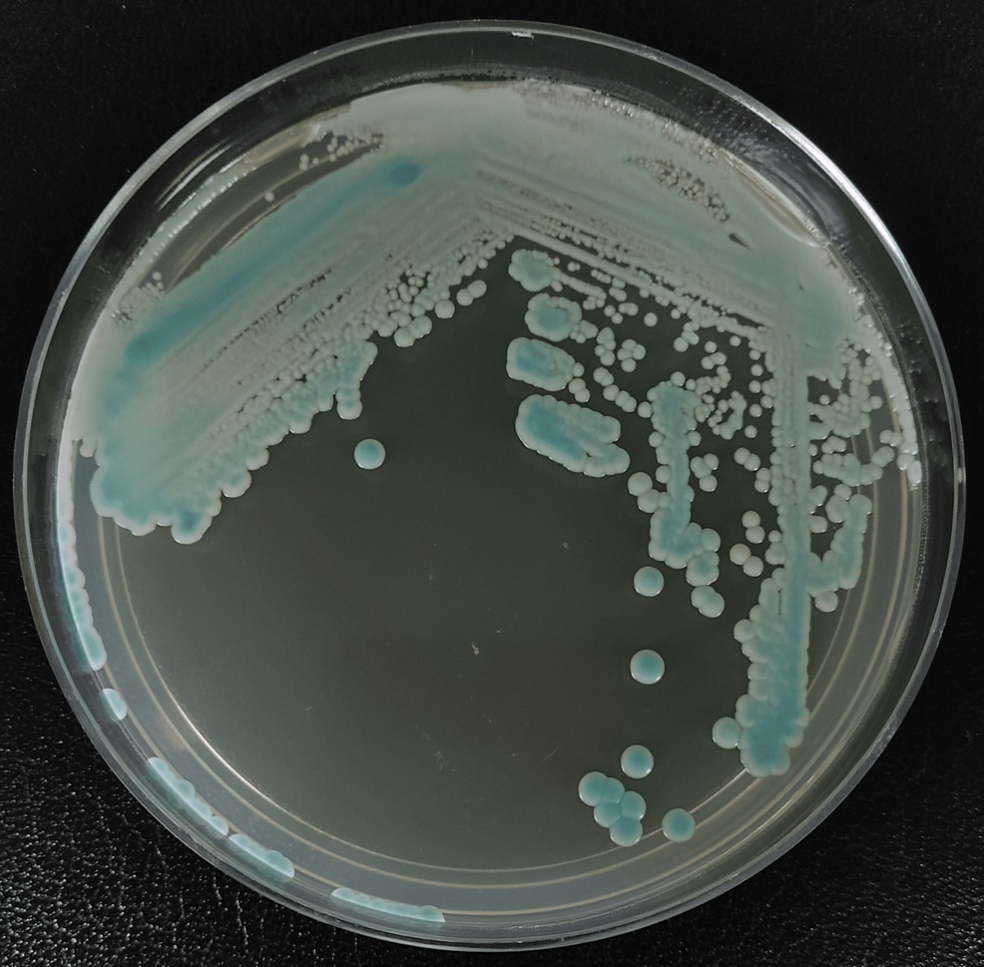
Candida chromogenic agar (HiCromeTM Candida) with turquoise blue colonies

**Figure 5:**
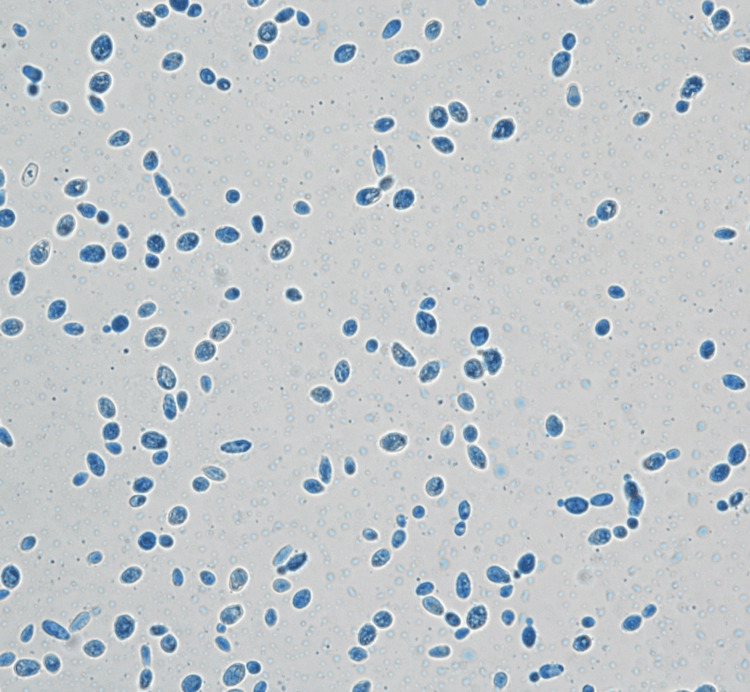
Microscopic view (×40) of yeast stained with Lactophenol Cotton Blue

**Figure 6:**
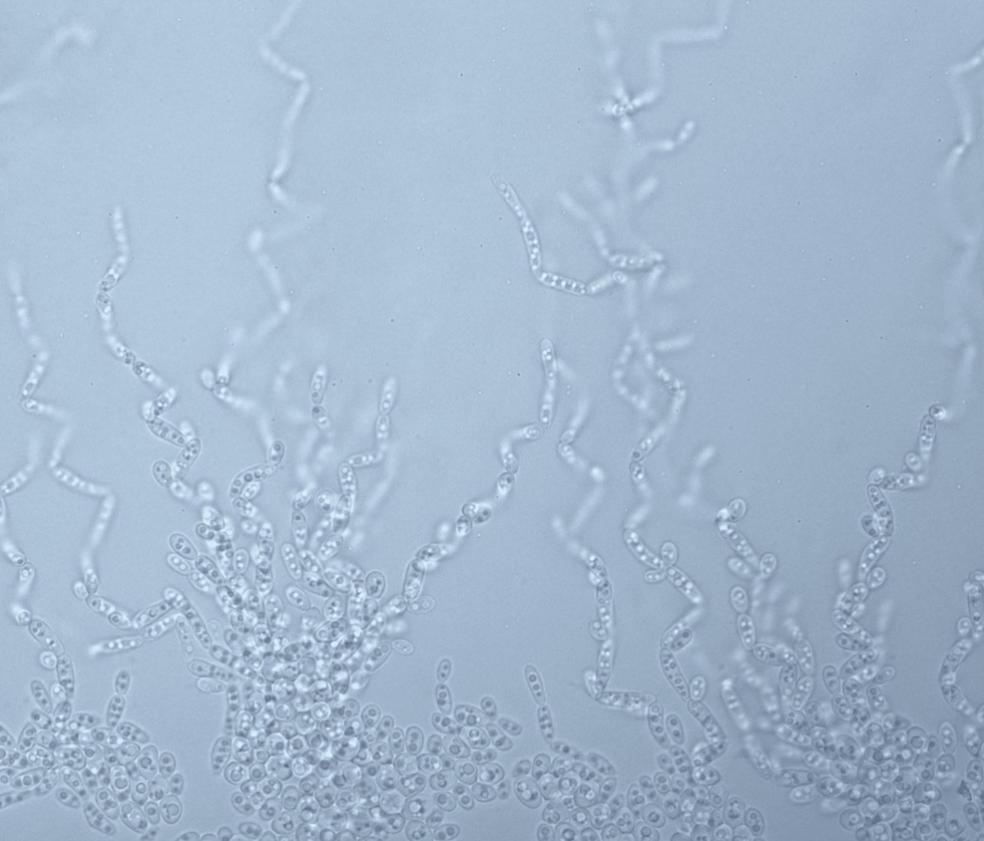
Microscopic view (×10) of slide culture showing pseudohyphae

*Lodderomyces elongisporus* was identified on day 14 of admission via matrix-assisted laser desorption/ionization time-of-flight (MALDI-TOF). At the same time, the isolate was sent to the Institute of Medical Research Malaysia for further analysis, where phenotypic and molecular studies were done. The primary cultures received were subcultured onto Sabouraud dextrose agar (SDA) and *Candida* chromogenic agar (HiCrome^TM^ Candida). Both agars were incubated at 36.5°C-37°C. Both cultures read after 48 hours revealed cream-colored colonies on SDA (Figure 3) and turquoise blue colonies on *Candida* chromogenic agar (Figure 4). Using light microscopy (Olympus CX43), microscopic features were examined by making a direct smear of the colonies and staining with Lactophenol Cotton Blue (LPCB) dye (Figure 5). The slide culture was also done and examined (Figure 6).

**Figure 7:**
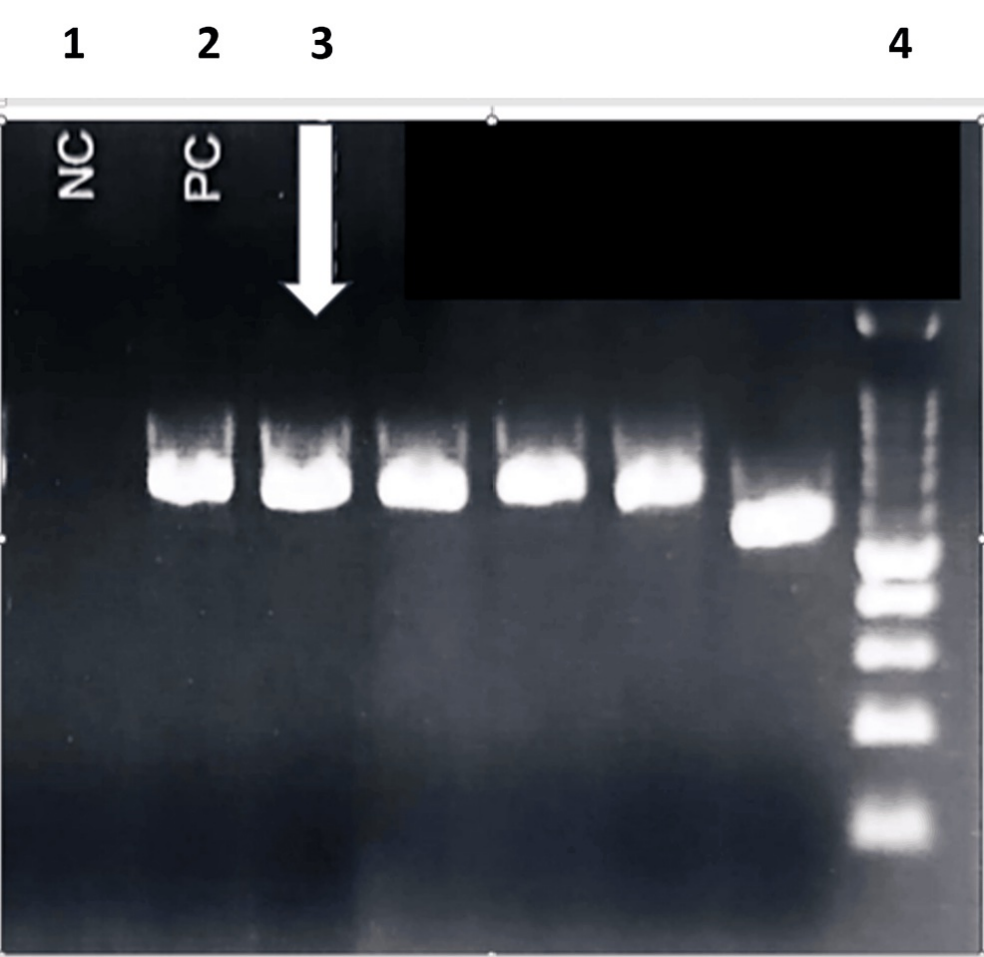
Agarose gel electrophoresis The representative gel picture showing RedSafe™ solution stained 700 bp amplified product of *Lodderomyces elongisporus* (lane 3). No band is shown at the negative control lane (lane 1). A single band is shown at the positive control lane with a PCR product size of around 800 bp (lane 2). 100 bp DNA ladder (lane 4). PCR: polymerase chain reaction, DNA: deoxyribonucleic acid

Specification of the organism was achieved by using DNA extraction and polymerase chain reaction (PCR) and DNA sequencing polymerase chain reaction. All chemicals and reagents used in the DNA extraction were purchased from Roche Diagnostics. The supernatant was added to the cartridge, and genomic DNA was extracted according to the manufacturer's instructions. Polymerase chain reaction and DNA sequencing polymerase chain reaction (PCR) were performed using primers NL1 and NL4 purchased from Integrated DNA Technologies, Inc. (IDT) (Coralville, IA, USA). A 1× PCR master mix was prepared as follows: 12.5 μL MyTaq HS Mix 2×, 0.5 μL each of the forward and reverse primers, 5 μL template DNA, and 6.5 μL molecular biology grade H2O. They were added to a total volume of 20 μL. Amplifications were carried out in thermocycler with the following thermal cycling parameters: initial denaturation at 95°C for one minute, followed by heat denaturation at 95°C for 15 seconds, annealing at 58°C for 15 seconds, and extension at 72°C for 10 seconds. The PCR products were visualized on 1.5% agarose gel after electrophoresis (Figure 7). PCR products (20 μL) were sent to a private laboratory for sequencing. The sequences were then identified using the Basic Local Alignment Search Tool (BLAST) program. Sequence-based identifications were defined by percent identity: species, ≥99%; genus, 94%-98%; and inconclusive, ≤93%. The strain was identified as *Lodderomyces elongisporus* (GenBank accession number: MK394128.1, percentage identity: 99.82%). This sequencing result of the culture isolate was consistent with the MALDI-TOF identification.

Antifungal susceptibility testing was done using YeastOne Sensititre to determine the minimum inhibitory concentrations (MICs) of several antifungals (Table 2). However, there is no established breakpoint for *Lodderomyces elongisporus* to interpret the susceptibility of the antifungals.

**Table 2:** Antifungal susceptibility testing MIC: minimum inhibitory concentration

Antifungal	MIC (µg/mL or mg/L)
5-Flucytosine	0.12
Amphotericin B	0.5
Anidulafungin	0.03
Caspofungin	0.06
Fluconazole	0.25
Itraconazole	0.06
Micafungin	0.03
Posaconazole	0.03
Voriconazole	<0.008

**Figure 8:**
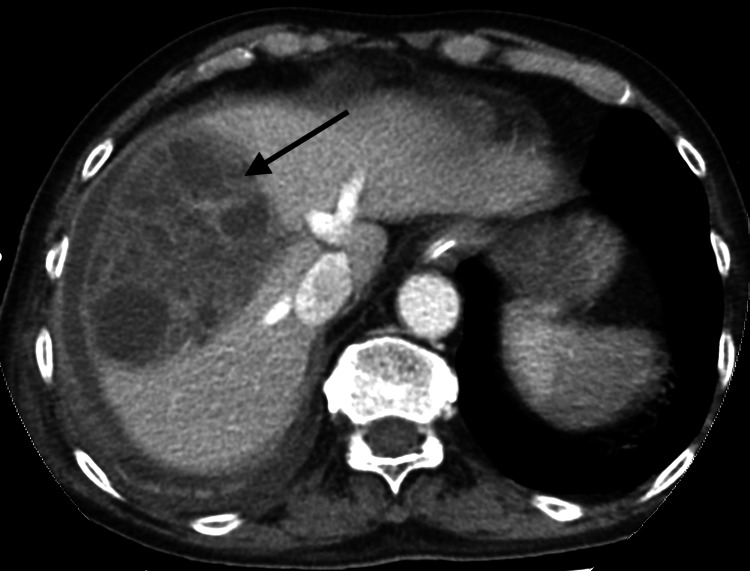
Axial view of computed tomography of the abdomen showing right liver lobe abscess (black arrow) with subcapsular liver collection

On day 15 of admission, a contrast-enhanced computed tomography of the abdomen confirmed a ruptured large right liver lobe abscess with subcapsular liver collection and reactive cholecystitis (Figure 8). An ultrasound-guided percutaneous drainage of the liver abscess and subphrenic/perihepatic collection was done the next day. The pus culture of the liver abscess showed mixed growth of at least three types of organisms, and the surveillance blood culture results showed *Staphylococcus haemolyticus *in which its significance was deemed doubtful*.*

Although his white blood cell and C-reactive protein reduced, his procalcitonin levels and lactate dehydrogenase levels continued to rise while on antibiotics and antifungal (Table 3). Eventually, he succumbed to the disease after three weeks of hospitalization.

**Table 3:** Trend of inflammatory markers during hospitalization

Inflammatory markers	Day 1	Day 3	Day 5	Day 8	Day 11	Day 14	Day 19
White blood cell (4-10 × 10³/µL)	28.7	28.1	25.5	18.2	18.7	18.5	10.3
C-reactive protein (0-0.5 mg/dL)	36.3	33	-	-	-	-	16.3
Lactate dehydrogenase (0-247 U/L)	1,048	813	-	-	555	1,091	-
Procalcitonin (0-0.5 ng/mL)	-	-	6.76	3.4	6.3	6.09	-

## Discussion

*Lodderomyces elongisporus* is a rare but emerging infection that should be taken note of, from being isolated in fruits and soft drinks [2] to being described as a fatal bloodstream infection [7]. To the best of our knowledge, 40 *Lodderomyces elongisporus *cases are reported worldwide, including our current case [5]. The majority of the cases occur in Asian countries, with India having the highest reported numbers to date [8,9]. In Malaysia, there are four cases before this, making this the fifth. Most cases are found in extreme age groups, specifically the elderly and infants [5]. On top of that, nearly all cases had underlying conditions, with four of them having malignancy [5], like in our patient. Solid tumor is one of the most common host risk factors for developing invasive fungal infections [10], with gastrointestinal tumors being the most frequent type [11]. Our patient had a pancreatic tumor that was radiologically suspicious but has not been confirmed histologically to be malignant. Unfortunately, he passed away before the pancreatic tumor could be removed and analyzed.

Although *Lodderomyces elongisporus* was isolated only after a week of hospitalization in our patient, we strongly feel that this could be a co-infection of both *Lodderomyces elongisporus* and *Pantoea dispersa* rather than this yeast being a hospital-acquired infection. This patient presented with septic shock and dissemination of the disease involving his lungs and liver. There have been a few reported cases of *Pantoea dispersa* infection, but none showing disseminated disease. It is worth noting that *Pantoea dispersa* is rarely pathogenic due to its lack of overtly invasive virulence factors and acts more like an endophyte [6]. The first reported case of *Pantoea dispersa* was found in an elderly lady with acute myeloid leukemia and multiple myeloma on palliative chemotherapy who was treated for pneumonia and discharged well after a course of antibiotics [12]. Other cases reported were mostly hospital-acquired, from a pseudo-outbreak of *Pantoea​​​​​​​ dispersa* joint infection due to a laboratory contaminant [13] to neonatal sepsis found in patients delivered in the same operating theater [14]. On the other hand, invasive fungal infection is commonly found in immunocompromised people [10]. The infection source in most *Lodderomyces elongisporus *cases was bloodstream infections with documented organ involvement such as endocarditis, osteomyelitis, and meningitis [15,16]. This raises suspicion that this yeast could have been responsible for causing the dissemination of the disease. Undeniably, the patient also had hospital-related risk factors for developing fungemia, which included central venous catheterization, prolonged intensive care unit stay, and usage of broad-spectrum antibiotics [10].

Out of the 39 reported cases of *Lodderomyces elongisporus*, eight resulted in death, giving a mortality rate of 20% [5]. Comorbidities such as heart disease, advanced malignancy, and end-stage renal failure were described in adult patients with fatal *Lodderomyces elongisporus* infection [5]. In addition to that, low birth weight and prematurity were among the risk factors that led to the demise of patients in the neonate group [5]. Our patient possibly had undiagnosed heart disease as evidenced by his reduced ejection fraction and atrial fibrillation, on top of being immunocompromised due to his pancreatic tumor. The disseminated disease could have worsened his pre-existing heart disease and contributed to his death. It is observed that the mortality rate of patients with invasive fungal infections is as high as 40% even with antifungal therapy [17]. This could be attributed to the lack of effectiveness of our diagnostic methods in detecting and identifying the yeast promptly for the initiation of treatment [17]. Studies have shown that both blood culture and sterile site culture have poor sensitivity in detecting fungal organisms due to the lack of viable organisms/low concentration of organisms in the site of the collected samples [17]. This was also reflected in our case whereby his initial blood culture on presentation, pleural fluid culture, and pus culture of the ruptured liver abscess were negative for the yeast. Non-culture tests such as polymerase chain reaction could have been performed for early detection of the yeast and to potentially alter the patient's outcome [17].

The next crucial step of the management is identifying the culprit yeast. CHROMagar is an important tool in resource-limited settings for yeast identification by recognizing *Candida* species based on the color of the colonies [18]. For example, green colonies are seen in *Candida albicans*, steel blue colonies with purple pigmentation are seen in *Candida tropicalis*, and cream-colored colonies with pink shades are seen in *Candida parapsilosis* [18]. The number of *Lodderomyces elongisporus* cases reported worldwide is sparse due to the frequent misidentification of the species using older yeast identification systems [2-4]. Early and accurate recognition of organisms plays a vital role in the management, but sadly, many conventional ways of yeast identification often take too long [19]. Although DNA sequencing is the gold standard for yeast identification, it is costly and requires trained personnel with proper software, making it not readily available in all clinical settings [19]. In recent years, MALDI-TOF has become a cost-friendly and efficient method for organism identification, by comparing the peptide mass fingerprint of the organism to the database [19]. Fortunately, every state hospital in Malaysia has MALDI-TOF as a form of yeast identification system to ease clinicians' management.

At the moment, there is no breakpoint for *Lodderomyces elongisporus* to interpret the susceptibility of the antifungals. However, the isolate shows low minimum inhibitory concentration to a wide range of antifungals [5]. The Infectious Disease Society of America (IDSA) guidelines recommend the usage of echinocandin for hemodynamically unstable patients with fungemia, as in our present case [20]. The use of echinocandins, azoles, and amphotericin B was described in previous case reports [5], but it was unclear as to which antifungal was most superior.

## Conclusions

There are many emerging rare yeast species nowadays with the advancement of medical treatment and increasing survival of critically ill patients. The delay in the identification of yeast and antifungal susceptibility testing often contributes to the morbidity and mortality of patients. Hence, it is important for effective communication among clinicians and microbiologists to improve patients' outcomes. Early referral to the infectious disease team is also highlighted for the appropriate choice of antifungal while awaiting the culture and sensitivity results.
